# How to Construct a Combined S-CO_2_ Cycle for Coal Fired Power Plant?

**DOI:** 10.3390/e21010019

**Published:** 2018-12-27

**Authors:** Enhui Sun, Han Hu, Hangning Li, Chao Liu, Jinliang Xu

**Affiliations:** 1 Beijing Key Laboratory of Multiphase Flow and Heat Transfer for Low Grade Energy Utilization, North China Electric Power University, Beijing 102206, China; 2 Key Laboratory of Condition Monitoring and Control for Power Plant Equipment of Ministry of Education, North China Electric Power University, Beijing 102206, China

**Keywords:** S-CO_2_ coal fired power plant, combined cycle, thermodynamics, exergy destruction, residual heat of flue gas

## Abstract

It is difficult to recover the residual heat from flue gas when supercritical carbon dioxide (S-CO_2_) cycle is used for a coal fired power plant, due to the higher CO_2_ temperature in tail flue and the limited air temperature in air preheater. The combined cycle is helpful for residual heat recovery. Thus, it is important to build an efficient bottom cycle. In this paper, we proposed a novel exergy destruction control strategy during residual heat recovery to equal and minimize the exergy destruction for different bottom cycles. Five bottom cycles are analyzed to identify their differences in thermal efficiencies (*η_th,b_*), and the CO_2_ temperature entering the bottom cycle heater (*T*_4*b*_) etc. We show that the exergy destruction can be minimized by a suitable pinch temperature between flue gas and CO_2_ in the heater via adjusting *T*_4*b*_. Among the five bottom cycles, either the recompression cycle (RC) or the partial cooling cycle (PACC) exhibits good performance. The power generation efficiency is 47.04% when the vapor parameters of CO_2_ are 620/30 MPa, with the double-reheating-recompression cycle as the top cycle, and RC as the bottom cycle. Such efficiency is higher than that of the supercritical water cycle power plant.

## 1. Introduction

Coal fired power generation accounts for nearly 32% of the world’s total electricity production [1]. However, this application is a concern due to environmental pollution and carbon dioxide emissions [2,3]. Thus, a clean, efficient, and flexible coal fired power plant is attractive for power generation. S-CO_2_ cycle has extensive application in power generation, such as concentrating solar power generation [4,5,6], nuclear power generation [7,8,9], waste heat utilization of gas turbine [10,11,12] due to its high efficiency [13,14], simple system [10,15], and compact layout [16,17]. These advantages make it attractive to apply a S-CO_2_ cycle to coal fired power generation, but this system faces a residual heat problem. 

The main reason for this problem is that the cycle has a good regenerative effect, leading to a high CO_2_ temperature at the boiler inlet. For example, recompression cycle is a commonly used S-CO_2_ cycle, when the double reheating recompression cycle is applied, the CO_2_ temperature at boiler inlet is about 510 °C [18,19]. While this temperature is about 340 °C in the double reheating supercritical water cycle [20]. The great temperature difference means a large amount of residual heat in the tail flue at S-CO_2_ coal fired power plant, which restricts the boiler efficiency. Therefore, how to solve this problem to get a high boiler efficiency is a key issue in this field. 

The solutions for this problem can be divided into three categories: (1) adding flue gas cooler [21,22,23], (2) raising air temperature [18,24,25], (3) constructing combined cycle [26,27,28]. 

The concept of adding flue gas cooler is to extract a part of low temperature CO_2_ flow rate from S-CO_2_ cycle into the tail flue to absorb residual heat [18]. It is found that thermal efficiency is decreased as increasing of the extracted flow rate since the additional heat is added to the cycle. However, adding flue gas cooler still has an efficiency benefit over no flue gas cooler due to lower compression work for the compressor system. 

Residual heat can be reduced by raising air temperature in air preheater. However, there are two limitations to this application. The first limitation is that the heat capacity of air is smaller than that of flue gas. Thus, with increase of air temperature, the temperature difference between flue gas and air decreases, leading to a sharply increased volume of air preheater. Another limitation is that the high-temperature air preheater is difficult to manufacture, thus a traditional tri-sector air preheater is not suitable for this case.

If we want to keep a high cycle thermal efficiency while maintain the air preheater compatible within the present engineering experience, constructing the combined cycle is a promising method. Johnson et al. [26] propose using a Rankine cycle to absorb residual heat. However, this scheme has lost some advantages of the S-CO_2_ cycle, such as simple system and compact layout. McClung et al. [27] suggest absorbing residual heat through two series recompression cycles. Both top and bottom cycles are S-CO_2_ cycles. Sun et al. [28] explore the power generation efficiency of applying different S-CO_2_ bottom cycles to absorb residual heat, such as a simple recuperated cycle, partial cooling cycle, recompression cycle, etc. It is found that the suitable bottom cycle is different for different turbine inlet temperatures. 

From the present research, it is shown that the analysis of combined cycle for S-CO_2_ coal fired power generation is based on the first law of thermodynamics. While we should pay more attention to the exergy destruction in residual heat transferring process to highlight the different bottom cycles. This is because the boiler efficiency is guaranteed when the residual heat is fully absorbed by the bottom cycle. However, exergy destruction of different bottom cycles in residual heat absorption process will be different, which restricts the comparison between different bottom cycles. In this paper, we attempt to answer the question that how to choose a highly efficient bottom cycle. This paper is organized as follows. Section 2 describes the mathematical model. Section 3.1 explains the causes of residual heat problem. Section 3.2 proposes the exergy destruction control strategy during residual heat recovery and explains the rationality of this strategy. In Section 3.3, five S-CO_2_ bottom cycles are analyzed to reveal their differences in thermal efficiency (*η_th,b_*), bottom cycle heater inlet temperature (*T*_4*b*_), etc. It is found that although the cycles are different, the strategy in Section 3.2 can be achieved by adjusting *T*_4*b*_. In Section 3.4, two kinds of better performance combined S-CO_2_ cycles are detailed analyzed in which the top cycle is double reheating recompression cycle (DRH), the bottom cycle is recompression cycle (RC), and partial cooling cycle (PACC), respectively.

## 2. System Description and Methods 

### 2.1. S-CO_2_ Cycle Description

**Figure 1:**
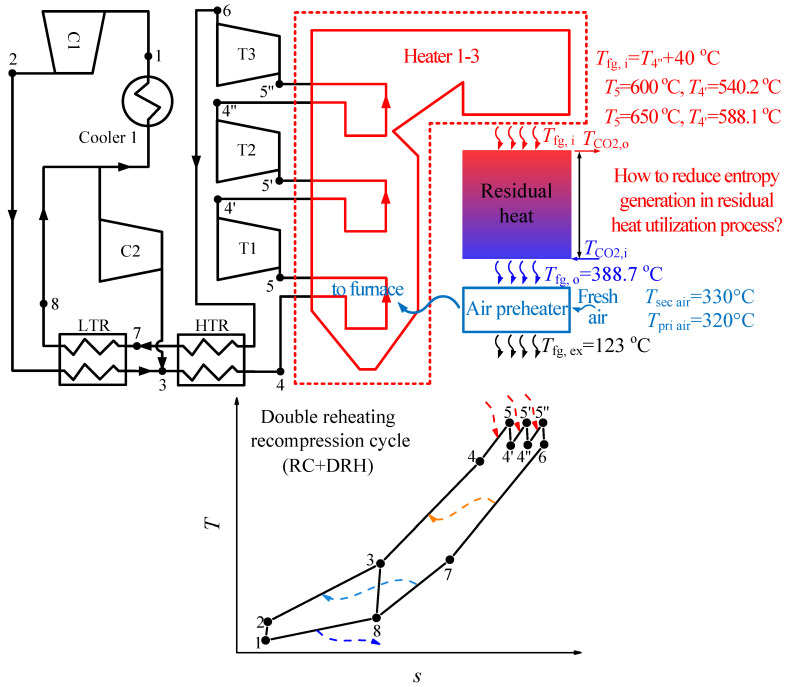
S-CO_2_ coal fired power generation system.

**Figure 2:**
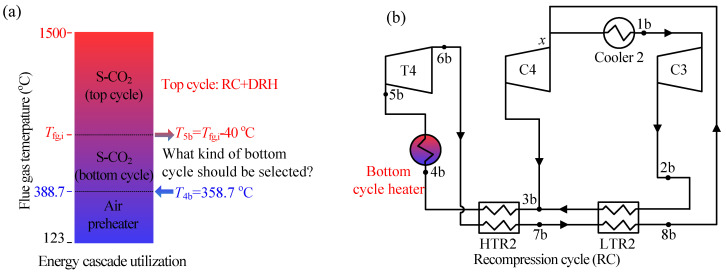
(**a**) Energy cascade utilization; (**b**) Layout of RC.

**Figure 3:**
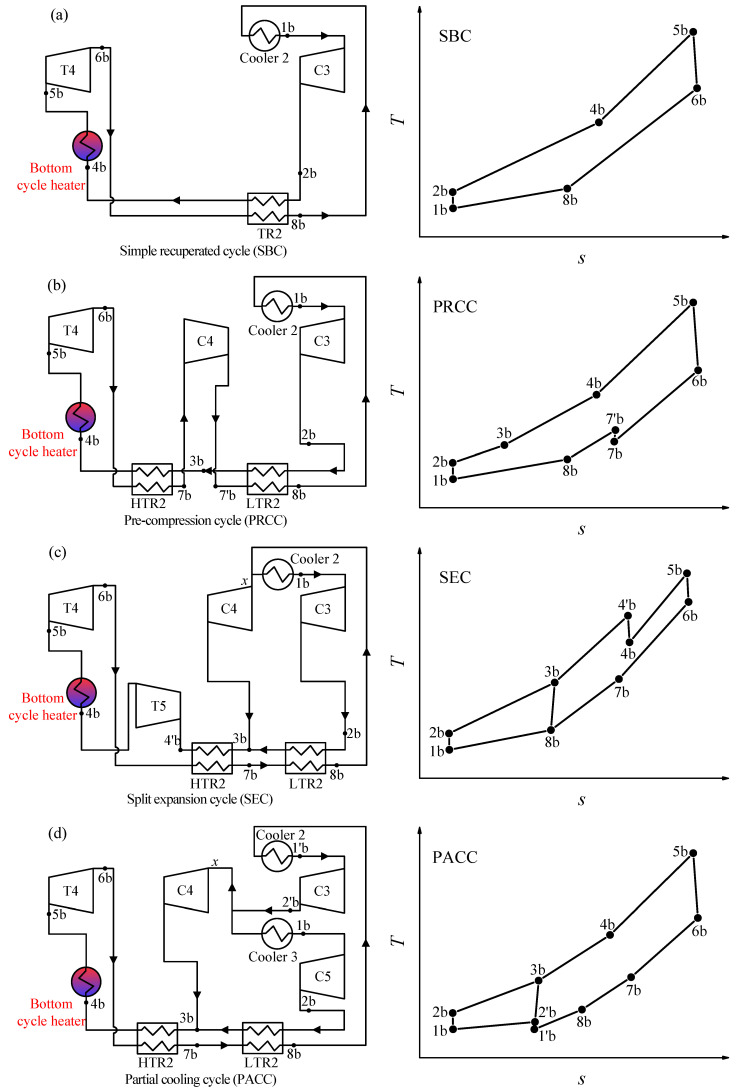
Four S-CO_2_ bottom cycles.

The combined cycle consists of the top cycle and bottom cycle. In this paper, the double reheating recompression cycle (RC + DRH) is selected as the top cycle, due to its high efficiency [18,24]. The flow diagram and *T*-*s* diagram of RC + DRH is shown in Figure 1. Five S-CO_2_ cycles are selected as the bottom cycle, such as simple recuperated cycle (SBC), pre-compression cycle (PRCC), recompression cycle (RC), split expansion cycle (SEC), partial cooling cycle (PACC), as shown in Figure 2 and Figure 3. Since there are many S-CO_2_ cycles involved, the characteristics of each cycle are introduced. 

For the top cycle, the CO_2_ flow stream after high-temperature recuperator (HTR) enters the boiler and converts thermal energy into power by three turbines (T1–T3). Then low-pressure CO_2_ from the outlet of T3 transmits heat to high-pressure CO_2_ through HTR and LTR (low-temperature recuperator). Here the following flow assignment should be highly focused due to the unique characteristics of the recompression cycle can be reflected by this layout: the flow from LTR is split into two streams, one stream needs to flow through cooler 1 into compressor 1 (C1). The other stream flows into the auxiliary compressor (C2), then enters the high-pressure side outlet of LTR (point 3). This flow assignment reduces the heat released into the environment by splitting part of CO_2_ directly into C2, which is an important reason for the high efficiency of recompression cycle. Table 1 summarized the parameters from the above calculation.

There are five kinds of bottom S-CO_2_ cycles, and a brief introduction is as follows:

Simple recuperated cycle (SBC): The SBC is one of the simplest S-CO_2_ cycles, shown in Figure 3a. There is only one regenerator. Due to the specific heat capacity difference between the cold and hot sides of the regenerator, the bottom cycle heater inlet temperature (*T*_4*b*_) is lower which leads to heat absorption in heat source, more under the unit mass flow rate.

Pre-compression cycle (PRCC): Different from SBC, the CO_2_ at the outlet of turbine 4 (T4) is at the subcritical state. This will increase the enthalpy drop in the turbine. Meanwhile, the regenerator is divided by two, and the subcritical state CO_2_ is compressed to the supercritical state by an additional compressor (C4), shown in Figure 3b. Although the outlet temperature of T4 decreased with the enthalpy drop of T4 increased, PRCC and SBC have similar bottom cycle heater inlet temperature (*T*_4*b*_). Thus, the heat absorption of both cycles is nearly the same, but more work is produced by PRCC, then the efficiency of PRCC is slightly higher than that of SBC (shown in Figure 5a,b).

Recompression cycle (RC): RC is the foundation of RC + DRH (see Figure 2b). It can be referred to the description of the top cycle.

Split expansion cycle (SEC): SEC is evolved by RC, shown in Figure 3c. The only difference is the bottom cycle heater is added between the turbine 4 (T4) and turbine 5 (T5). Thus, the inlet temperature of T5 is lower, leading to a lower thermal efficiency than RC (see Figure 5a).

Partial cooling cycle (PACC): PACC has the characteristics of PRCC and RC, shown in Figure 3d. First, the outlet parameter of turbine 4 (T4) is the subcritical state, similar to PRCC. Secondly, the CO_2_ flow rate is divided before entering the cooler 3, similar to RC. Thus, the efficiency of PACC is somewhere between PRCC and RC (see Figure 5a).

Table 2 summarized the calculated parameters for the bottom cycle.

### 2.2. Thermodynamic Model for S-CO_2_ Cycle

Different cycles have different characteristics. However, all of them can be simulated by the classical method. The following shows the simulation method based on RC for per unit total mass flow rate.

Isentropic efficiency and power output for turbine 4 (T4) are

(1) $$\eta_{T4,s}=\frac{h_{5b}-h_{6b}}{h_{5b}-h_{6b,s}},\;w_{T4}=h_{5b}-h_{6b}$$

Isentropic efficiency and power output for C3/C4 are

(2) $$\eta_{C3,s}=\frac{h_{2b,s}-h_{1b}}{h_{2b}-h_{1b}},\;w_{C3}=\left(1-x_{b}\right)\left(h_{2b}-h_{1b}\right)$$

(3) $$\eta_{C4,s}=\frac{h_{8b,s}-h_{3b}}{h_{8b}-h_{3b}},\;w_{C4}=x_{b}\left(h_{3b}-h_{8b}\right)$$

In Equations (1)–(3), the subscripts *s* represents isentropic condition, *x_b_* is the mass flow fraction, which is defined as the ratio of mass flow rate in C4 to total mass flow rate, and *h* stands for enthalpy. 

Energy conservation equation for LTR and HTR are given by

(4) $$h_{7b}-h_{8b}=\left(1-x_{b}\right)\left(h_{3b}-h_{2b}\right)$$

(5) $$h_{4b}-h_{3b}=h_{6b}-h_{7b}$$

The heat absorption by bottom cycle heater (*q_rb_*) and heat dissipated by cooler 2 (*q_c_*_2_) are

(6) $$q_{rb}=h_{5b}-h_{4b}$$

(7) $$q_{c2}=\left(1-x_{b}\right)\left(h_{8b}-h_{1b}\right)$$

The cycle thermal efficiency is

(8) $$\eta_{th,b}=\frac{w_{T4}-w_{C3}-w_{C4}}{q_{rb}}$$

### 2.3. Calculation Method of Residual Heat

In respect of flue gas energy distribution, the high temperature region is absorbed by top cycle, middle temperature region (residual heat region) is absorbed by bottom cycle, low temperature region is absorbed by air preheater (see Figure 2a). For top cycle and air preheater, when the total output power and air temperature are fixed, the amount of heat absorbed from the boiler is determined. Then, the remaining heat in boiler is the residual heat, which should be absorbed by the bottom cycle.

The boiler efficiency (*η_b_*) is calculated as [29]

(9) $$\eta_{b}=1-\sum_{i=2}^{6}q_{i}/100$$

where *q_i_* is the heat loss. For large-scale coal fired boiler, except *q*_2_ (exhaust gas heat loss), the other heat losses can be set as constants where *q*_3_ = 0 (incomplete chemical combustion heat loss), *q*_4_ = 0.6 (unburned carbon heat loss), *q*_5_ = 0.2 (furnace exterior heat transfer loss), *q*_6_ = 0.06 (enthalpy variation loss of ash and slag). *q*_2_ is calculated as

(10) $$q_{2}=\frac{\left(h_{fg,ex}-\alpha_{air}h_{air}\right)\left(1-q_{4}/100\right)}{Q_{LHV}}\times 100$$

where *α_air_* is the excess air ratio, *h_air_* is the air enthalpy at environment temperature. *Q_LHV_* is the lower heating value of coal (*Q_LHV_* = 23442 kJ/kg, see Table 3 for coal parameters).

When assuming the exhaust gas temperature (*T_fg,ex_*) is equal to the gas temperature at the bottom cycle heater inlet *(T_fg,i_)*, *T_fg,ex_* = *T_fg,i_* (see Figure 1), the equivalent boiler efficiency at bottom cycle heater inlet (*η_b,fg,i_*) can be calculated. Then, the coal consumption (*m_coal_*) is

(11) $$m_{coal}=\frac{Q_{rt}}{\eta_{b}Q_{LHV}}$$

where *Q_rt_* is the heat absorbed by heater 1–3.

The residual flue gas heat is calculated as

(12) $$Q_{rb}=m_{fg}\left(h_{fg,i}-h_{fg,o}\right)$$

where *m_fg_* is the mass flow rate of flue gas, which is the sum of coal consumption *m_coal_* and the mass flow rate of air *m_air_* (*m_fg_* = *m_coal_* + *m_air_*). Based on the *Q_rb_*, the mass flow rate of bottom cycle can be calculated according to Equation (6). Then the actual boiler efficiency can be calculated according to the set *T_fg,ex_* = 123 °C. The power generation efficiency *η_e_* is

(13) $$\eta_{e}=\eta_{b}\eta_{th}\eta_{p}\eta_{g}$$

where *η_p_* is the pipeline efficiency (evaluates the heat loss through the pipeline between each component) [30,31] and *η_g_* is the generator efficiency (see Table 1 for the values). The simulation in this paper is developed using FORTRAN language. Physical properties of CO_2_ come from REFPROP [32].

## 3. Results and Discussion

### 3.1. Causes of Residual Heat Problem

S-CO_2_ coal fired power generation system is shown in Figure 1. There is a large amount of residual heat in the tail flue. The residual heat is caused by two effects:

(1) The CO_2_ temperature is high in the tail flue: differences of heating surface layout between this paper and [28] is that in this paper the preheater in tail flue is eliminated. This change means that the CO_2_ at the outlet of HTR (point 4) enters the furnace directly rather than the tail flue. Thus, one of the reheater inlet temperature (*T*_4′_, *T*_4″_) is the lowest CO_2_ temperature in the tail flue. *T*_4′_ and *T*_4″_ can be calculated by the following equation

(14) $$\eta_{T1,s}=\frac{h_{5}-h_{4\prime }}{h_{5}-h_{4\prime ,s}},\;\;\eta_{T2,s}=\frac{h_{5\prime }-h_{4\prime \prime }}{h_{5\prime }-h_{4\prime \prime ,s}}$$

where *η_T_*_1,*s*_ and *η_T_*_2,*s*_ are the turbine isentropic efficiency, *h* is the enthalpy. To solve the above equation, it is also necessary to know the turbine inlet pressure [18]

(15) $$P_{5\prime }=\sqrt[{3}]{P_{5}^{2}P_{6}},\;P_{5\prime \prime }=\sqrt[{3}]{P_{5}P_{6}^{2}}$$

Based on Equations (14) and (15), *T*_4′_, *T*_4″_ can be calculated. For example, when *T*_5_ = 600 °C, *P*_5_ = 30 MPa, *T*_4′_ = 540.2 °C, *T*_4″_ = 542.8 °C, and *T*_5_ = 650 °C, *P*_5_ = 30 MPa, *T*_4′_ = 588.1 °C, *T*_4″_ = 590.4 °C. Therefore, the lowest CO_2_ temperature (*T*_4′_, *T*_4″_) in the tail flue is much higher than the lowest water temperature for the water-steam Rankine cycle which is usually around 340 °C [20]. Here *T*_4′_ is considered as the lowest CO_2_ temperature in the tail flue. When *T*_4′_ is obtained, the flue gas temperature after the S-CO_2_ cycle (*T_fg,i_*) can be calculated according to the pinch temperature (Δ*T_p,fg,i_*) between the flue gas and CO_2_ at point 4′

(16) $$T_{fg,i}=T_{4\prime }+\Delta T_{p,fg,i}$$

where Δ*T_p,fg,i_* = 40 °C in this work. Based on Equation (16), when *T*_4′_ = 540.2 °C, *T_fg,I_* = 580.2 °C, and *T*_4′_ = 588.1 °C, *T_fg,I_* = 628.1 °C.

(2) The air temperature is limited in the air preheater: tri-sector regenerative air preheater is widely used in large-scale coal fired power system. In the air preheater, flue gas heat is absorbed by primary air and secondary air. However, considering the cost and security issues [33], air temperature cannot be increased without limitation. In this paper the primary air temperature (*T_pri air_*) is set at 320 °C, the secondary air temperature (*T_sec air_*) is set at 330 °C, which is in line with existing engineering experience. Based on above assumption and other parameters shown in Table 1, the flue gas temperature at the inlet of air preheater can be determined by energy conservation equation

(17) $$Q_{flue\;gas}=Q_{pri\;air}+Q_{sec\;air}$$

where *Q_flue gas_* is the heat released by flue gas, *Q_pri air_, Q_sec air_* are the heat absorbed by primary air and secondary air. The heat of flue gas and air can be calculated by the temperature difference

(18) $$Q_{flue\;gas}=m_{fg}c_{p,fg}\left(T_{fg,o}-T_{fg,ex}\right)$$

where *m_fg_* is the mass flow rate of flue gas, *T_fg,o_* is the flue gas temperature at the inlet of air preheater, *c_p,fg_* is the heat capacity of flue gas, *T_fg,ex_* is the exhaust temperature of flue gas.

(19) $$Q_{pri\;air}=a_{pri}m_{air}c_{p,air}\left(T_{pri\;air}-T_{priair,in}\right)$$

where *α_pri_* is the primary air flow rate ratio, *T_pri air,in_* is the primary air temperature at the inlet of air preheater.

(20) $$Q_{sec\;air}=\alpha_{sec}m_{air}c_{p,air}\left(T_{sec\;air}-T_{sec\;air,in}\right)$$

where *α_sec_* is the secondary air flow rate ratio, *T_sec air,in_* is the secondary air temperature at the inlet of air preheater. 

*T_fg,o_* can be obtained from Equations (17)–(20) which is 388.7 °C. This temperature is far below *T_fg,i_* which are 580.2 °C and 628.1 °C when turbine inlet temperatures (*T*_5_) are 600 °C and 650 °C, respectively. The great temperature difference between *T_fg_*_,*i*_ and *T_fg_*_,*o*_ reflects that there is a lot of residual heat in the tail flue. If the residual heat cannot be efficiently absorbed, the boiler efficiency will be reduced due to the extra heat is discharged into the environment without being used. This problem can be called residual heat problem. Thus, how to efficiently solve this problem is a key issue for S-CO_2_ coal fired power generation. In this paper, we present a preliminary analysis solving this problem by constructing the combined cycle from the perspective of reducing exergy destruction of the heat transfer. The detailed analysis is shown in Section 3.2.

### 3.2. Exergy Destruction Control Strategy during Residual Heat Recovery

The combined cycle is constructed where the top cycle is the double reheating recompression cycle and the bottom cycle is the five different S-CO_2_ cycles (absorbing residual heat). Here, not only do we want the residual heat can be absorbed, but we also hope that the exergy destruction in residual heat absorption process is equal and minimum when comparing different bottom cycles. Exergy destruction (*I*) in the heat transfer process is

(21) $$I=T_{0}\int_{0}^{Q_{0}}\left(\frac{1}{T_{CO2}}-\frac{1}{T_{fg}}\right)dQ$$

where *T*_0_ is the environment temperature, *Q*_0_ is the heat transfer quantity between the flue gas and CO_2_, *T_fg_*, *T_CO2_* is the flue gas and CO_2_ temperature. Exergy destruction is caused by the temperature difference between flue gas and CO_2_. The above relationship also can be expressed by the integrated-average temperature difference (Δ*T_ave_*)

(22) $$\Delta T_{ave}=\frac{\int_{0}^{Q}(T_{fg}-T_{CO2})dQ}{Q_{0}}$$

**Figure 4:**
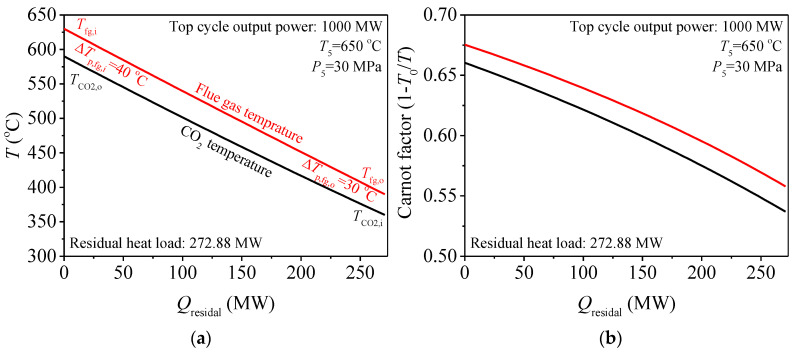
Exergy destruction control strategy during residual heat recovery. (**a**) *T*-*Q* chart for bottom cycle heater; (**b**) Carnot factor versus *Q* diagram for bottom cycle heater.

Based on the analysis in [34], *I* and Δ*T_ave_* share an exact linear relationship. Therefore, the exergy destruction in the heat transfer process is reduced to reduce the heat transfer temperature difference. In Equation (22), the $\int_{0}^{Q}(T_{fg}-T_{CO2})dQ$ represents the enclosed area formed by the flue gas temperature and CO_2_ temperature curves shown in Figure 4a. Thus, the effective way to reduce exergy destruction is to reduce the integrated-average temperature difference which is directly proportional to the enclosed area in *T*-*Q* chart. In Section 3.1, it is found that the inlet and outlet flue gas temperature of the bottom cycle heater (*T_fg,i_*, *T_fg,o_*) can be obtained. Meanwhile, in bottom cycle heater, the specific heat capacity of flue gas and CO_2_ is nearly constant, because the operation parameters are far from the critical region. Under such conditions, it is suggested that the CO_2_ temperature is to approach flue gas temperature to reduce the enclosed area. However, this approach should be limited, to keep a reasonable heat exchanger area. Here the pinch temperature at the inlet and outlet of bottom cycle heater are set: *ΔT_p,fg,I_* = 40 °C, *ΔT_p,fg,o_* = 30 °C. This matching relationship between flue gas and CO_2_ ensures the efficient utilization of residual heat which can be called the exergy destruction control strategy during residual heat recovery. The Carnot factor versus residual heat diagram is also used to illustrate the exergy destruction due to the heat transfer, shown in Figure 4b. The area between the curves in diagram represents the exergy destruction [35,36,37]. It is shown that, under such strategy, exergy destruction can be uniformly distributed and controlled. The next step is to explore what kind of S-CO_2_ cycle is suitable as the bottom cycle for S-CO_2_ coal fired power generation system based on this strategy. 

### 3.3. Analysis of Five S-CO_2_ Bottom Cycle

Five S-CO_2_ cycles have been introduced in Section 2.1 (shown in Figure 2 and Figure 3). In this section, these S-CO_2_ cycles are explored to reveal which is the most suitable as the bottom cycle when ensuring the exergy destruction control strategy during residual heat recovery. Here, thermal efficiency of bottom cycle (*η_th,b_*) and CO_2_ temperature at the inlet of bottom cycle heater (*T*_4*b*_) should be highly concerned. 

**Figure 5:**
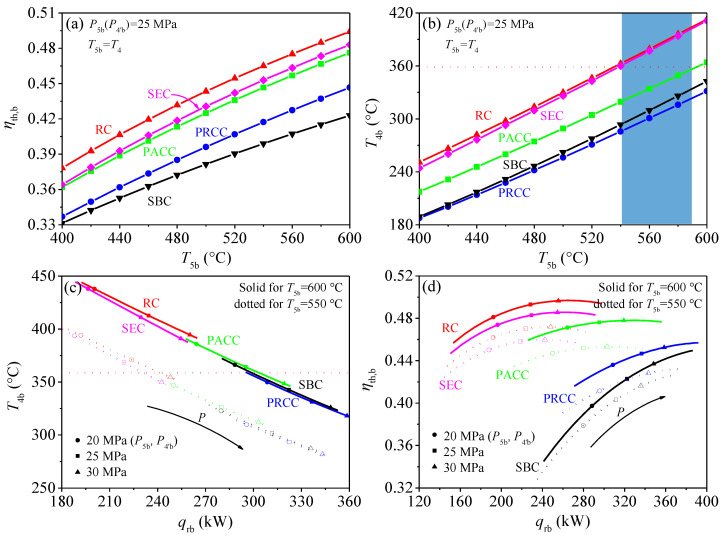
Characteristics of five S-CO_2_ bottom cycles. (**a**) relationship between the thermal efficiency (*η_th,b_*) and turbine inlet temperature (*T*_5*b*_) for bottom cycle; (**b**) relationship between the bottom cycle heater inlet temperature (*T*_4*b*_) and turbine inlet temperature (*T*_5*b*_); (**c**) relationship between the bottom cycle heater inlet temperature (*T*_4*b*_) and heat absorption per unit mass flow by bottom cycle heater (*q_rb_*); (**d**) relationship between the thermal efficiency (*η_th,b_*) and heat absorption per unit mass flow by bottom cycle heater (*q_rb_*).

Different S-CO_2_ cycles have different thermal efficiency: A higher *η_th,b_,* means more work is transformed by residual heat and less heat is released into the environment, due to the total amount of residual heat is fixed. From Figure 5a, it is shown that, under the design condition (see Table 2), the thermal efficiency of the recompression cycle (RC) is better than that of other S-CO_2_ cycles. The differences of *η_th,b_* among different S-CO_2_ cycles is very obvious. For example, when *T*_5*b*_ = 600 °C, *P*_5*b*_ = 25 MPa, the *η_th,b_* of RC is 49.41%, which is 1.80% higher than the *η_th,b_* of PACC and 7.12% higher than the *η_th,b_* of SBC. A question is driven by such a great difference in *η_th,b_*: if RC is the best cycle as the bottom cycle? The answer is no, because, besides the bottom cycle efficiency, the exergy destruction of residual heat should also be considered.

The exergy destruction of residual heat is decided by *T*_4*b*_: Another key parameter is *T*_4*b*_, the matching relationship between flue gas and CO_2_ is determined by *T*_4*b*_. From Figure 4a it can be seen that the exergy destruction in bottom cycle heater can be related to four parameters (*T_CO2,o_, T_fg,i_,* *T_fg,o_, T_CO2,i_*). When the residual heat is absorbed by bottom cycle, *T_CO2,o_* = *T*_5*b*_, *T_CO2,I_* = *T*_4*b*_. Among the four parameters, *T*_5*b*_ can be solved by top cycle. *T_fg,i_* is connected with *T*_5*b*_ according to *ΔT_p,fg,i_.* *T_fg,o_* can be calculated from the energy conservation equation in air preheater. Thus, the exergy destruction of residual heat is mainly decided by *T*_4*b*_. However, different S-CO_2_ cycles have different *T*_4*b*_. If we want to keep a smaller exergy destruction, that is to ensure the relationship of *T_fg,I_ = T_CO2,o_* + 30 °C, then *T*_4*b*_ should be kept at 358.7 °C. As shown in Figure 5b, *T*_4*b*_ of RC and SEC is higher than this constant value under the suitable operating temperature range of *T*_5*b*_ (see light blue area), and *T*_4*b*_ of the other cycles are lower than this constant value. Thus, *T*_4*b*_ should be adjusted to meet the exergy destruction control strategy during residual heat recovery.

*T*_4*b*_ can be affected by many variables, such as turbine and compressor isentropic efficiency, regenerator pinch temperature, cooler outlet, and turbine inlet parameters. However, isentropic efficiency and pinch temperature are restricted by the design and manufacture level of components. The cooler outlet parameters should be close to the critical point, to lower the compressor power. Therefore, *T*_4*b*_ is actually affected by turbine inlet parameters. For turbine inlet parameters (*T*_5*b*_, *P*_5*b*_), *T*_5*b*_ is determined by top cycle, so turbine inlet pressure (*P*_5*b*_) is needed to be highly concerned. Figure 5c shows the relationship between *T*_4*b*_ and *P*_5*b*_, it can be seen that with increase of *P*_5*b*_, *T*_4*b*_ can be effectively reduced. Meanwhile, the heat absorbed by the bottom cycle heater (*q_rb_*) is increased due to the temperature difference of *T*_5*b*_ and *T*_4*b*_ is increased. According to this feature, *T*_4*b*_ of all S-CO_2_ cycles can be adjusted to a certain value by adjusting *P*_5*b*_ to realize a better matching between the flue gas and CO_2_ in bottom cycle heater.

Based on the above analysis, there is no need to have a further analysis of some S-CO_2_ cycle owing to their poor performance, such as SBC and PACC. For both of them, in order to maintain *T*_4*b*_ = 358.7 °C, *P*_5*b*_ should be decreased. With the decrease of *P*_5*b*_, the thermal efficiency of SBC and PRCC is sharply reduced as shown in Figure 5d. For example, PACC and PRCC have an intersection point with the line of *T*_4*b*_ = 358.7 °C respectively when *T*_5*b*_ = 600 °C, shown in Figure 5c. The intersection point represents the exergy destruction satisfying the exergy destruction control strategy, shown in Figure 4. Under such circumstance, the *η_th,b_* (PRCC) = 42.96%, the *η_th,b_* (PACC) = 47.67% shown in Figure 5d. The huge efficiency difference means that while absorbing the same residual heat, PACC transformed more work. Due to all output work is exergy, thus, compared with PRCC, PACC not only reduces the exergy destruction in the process of absorbing residual heat but also converts the absorbed residual heat into more exergy. The same method can be applied to the comparison between PACC and SBC. The results show that the efficiency of PACC is 6.72% higher than that of SBC. So, based on the above analysis, performance of PRCC and SBC is inferior to that of PACC. Meanwhile, another comparison process is performed between the SEC and RC. *T*_4*b*_ is similar between SEC and RC, but the efficiency of RC is higher than that of SEC. Thus, from the perspective of thermal efficiency, RC is better than SEC. Finally, the PACC and RC are selected as the promising bottom cycle to have a further analysis.

### 3.4. Analysis of Two Combined S-CO_2_ Cycles

**Figure 6:**
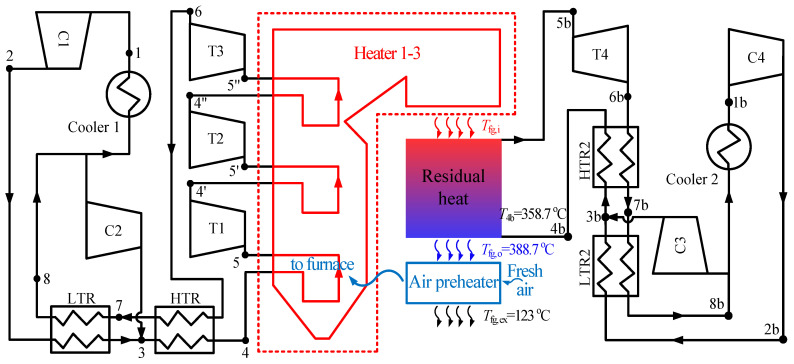
Combined cycle of double reheating recompression cycle + recompression cycle (RC + DRH + RC).

**Figure 7:**
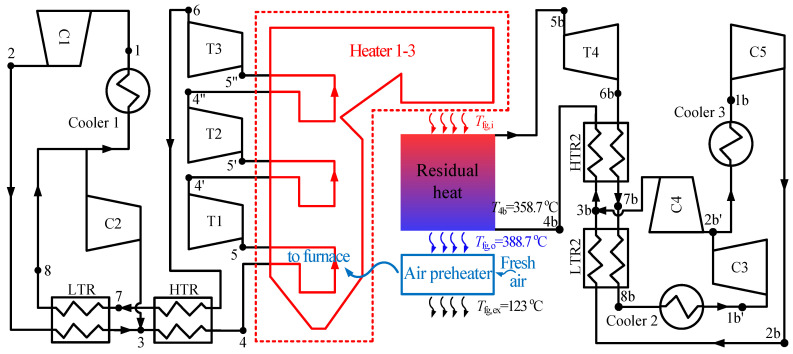
Combined cycle of double reheating recompression cycle + partial cooling cycle (RC + DRH + RC).

Two combined S-CO_2_ cycles have been constructed as shown in Figure 6 and Figure 7. The first combined cycle is called as RC + DRH + RC. RC + DRH is the top cycle which means double reheating recompression cycle, RC is the bottom cycle which means recompression cycle. The second combined cycle is called as RC + DRH + PACC. Compared with RC + DRH + RC, the difference is the bottom cycle changed from RC to PACC. 

**Figure 8:**
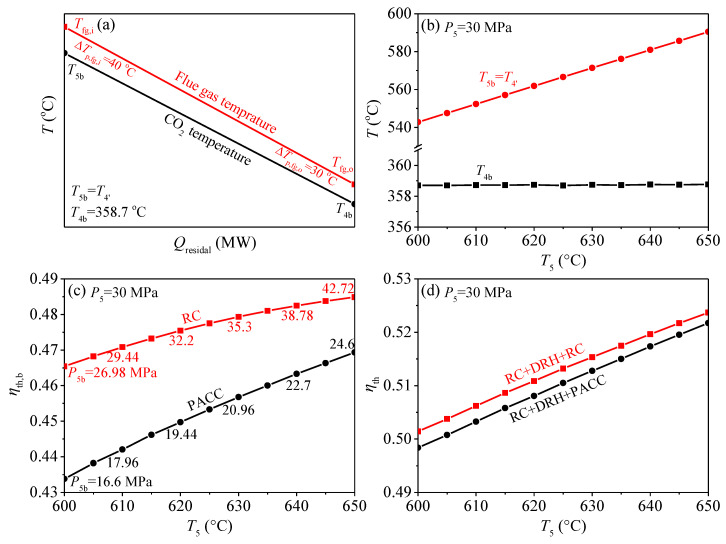
Characteristics of two combined S-CO2 cycles. (**a**) *T*-*Q* chart for bottom cycle heater; (**b**) variation of the bottom cycle heater inlet and outlet temperature (*T*_4*b*_, *T*_5*b*_) with top cycle turbine inlet temperature (*T*_5_); (**c**) relationship between the bottom cycle thermal efficiency (*η_th,b_*) and top cycle turbine inlet temperature (*T*_5_); (**d**) relationship between the combined cycle thermal efficiency (*η_th_*) and top cycle turbine inlet temperature (*T*_5_).

In this section, the exergy destruction control strategy during residual heat recovery is also ensured, shown in Figure 8a. Under this condition, the characteristics of the two combined cycles are compared. Figure 8b shows the relationship between some key point temperatures (*T*_5*b*_, *T*_4′_, *T*_4*b*_) and the turbine inlet temperature (*T*_5_). With increase of *T*_5_, *T*_5*b*_ increases, meanwhile, *T*_4*b*_ remains unchanged. Keeping *T*_4*b*_ constant means that the exergy destruction of the residual heat is controlled in the heat transfer process. In order to ensure *T*_4*b*_ unchanged, *P*_5*b*_ should be increased, and *q_rb_* increased with *P*_5*b*_ increased, then the residual heat is effectively absorbed. Temperature relationships in Figure 8b do not distinguish which bottom cycle to apply, it can be considered as the computing boundary condition for the bottom cycle. The calculation results for two combined cycles are shown in Figure 8c,d. Figure 8c shows the bottom cycle thermal efficiency of RC and PACC. To keep the exergy destruction control strategy during residual heat recovery, *P*_5*b*_ of RC and PACC is increased. Under such condition, the bottom cycle thermal efficiency of RC is higher than that of PACC. The final combined cycle thermal efficiency is shown in Figure 8d. Similarly, the thermal efficiency of RC + DRH + RC is higher than that of RC + DRH + PACC. Can such a result indicate that the RC should be selected as the bottom cycle? It still cannot come to such a conclusion. To answer this question, Figure 8c should be reviewed again. Figure 8c not only shows the thermal efficiency of the two bottom cycles, but also lists the needed *P*_5*b*_ to ensure *T*_4*b*_ = 358.7 °C. It is shown that *P*_5*b*_ increases with *T*_5_. When *T*_5_ increased from 600 °C to 650 °C, in order to fully absorb residual heat, *P*_5*b*_ increased from 26.98 MPa to 42.72 MPa for RC, and 16.6 MPa to 24.6 MPa for PACC. High pressure of *P*_5*b*_ for RC has exceeded engineering experience, such as 42.72 MPa. Therefore, the application of two combined cycles can be divided according to the limitation of materials. For example, when *T*_5_ = 620 °C, the thermal efficiency and power generation efficiency of RC + DRH + RC are 51.08% and 47.04% respectively. When *T*_5_ = 650 °C, the thermal efficiency and power generation efficiency of RC + DRH + PACC are 52.17% and 48.04% respectively. This result reflects the efficiency benefits of the combined S-CO_2_ cycle. Thus, even though the proposed cycle is complex, it is still necessary for the field of S-CO_2_ coal fired power generation. We are also looking for methods to simplify the power system to make it easier to install and manage.

## 4. Conclusions

The residual heat problem is one of the major issues for limiting the application of S-CO_2_ cycle in coal fired power generation. In this paper, the combined cycle is constructed to solve this problem. The main conclusions are drawn as follows:

1. The exergy destruction control strategy during residual heat recovery is proposed which can be set as the boundary condition for different bottom cycles comparison. The purpose of this strategy is to ensure that the exergy destruction in residual heat absorption process is equal and minimum when comparing different bottom cycles.
2. Five S-CO_2_ bottom cycles are simulated. It is shown that different S-CO_2_ cycles exhibit different characteristics. In order to ensure the exergy destruction control strategy during residual heat recovery, CO_2_ temperature at the inlet of bottom cycle heater (*T*_4*b*_) should be adjusted by tuning the turbine inlet pressure of bottom cycle (*P*_5*b*_).
3. When the top cycle is a double reheating recompression cycle (RC + DRH), the recompression cycle (RC) and partial cooling cycle (PACC) are suitable as the bottom cycle due to their better performance. Meanwhile, the RC + DRH + RC and RC + DRH + PACC are suitable for different temperature regions. It is recommended that when turbine inlet temperature of top cycle (*T*_5_) is 600–630 °C, RC + DRH + RC is more suitable, when *T*_5_ is 630–650 °C, RC + DRH + PACC should be proposed.

## Figures and Tables

**Table 1:** S-CO_2_ top cycle parameters and energy distribution in boiler.

Parameters	Values
Turbine inlet temperature (*T*_5_)	600–650 °C
Turbine inlet pressure (*P*_5_)	30 MPa
Turbine isentropic efficiency (*η_t,s_*)	93%
Compressor C1 inlet temperature (*T*_1_)	32 °C
Compressor C1 inlet pressure (*P*_1_)	7.6 MPa
Compressors isentropic efficiency (*η_c,s_*)	89%
Pressure drops in LTR and HTR (*ΔP*)	0.1 MPa
LTR and HTR pinch temperature difference (*ΔT_LTR_* or *ΔT_HTR_*)	10 °C
Primary air temperature (*T_pri air_*)	320 °C
Primary air temperature at the inlet of air preheater (*T_pri air,in_*)	31 °C
Primary air flow rate ratio (*α_pri_*)	19%
Secondary air temperature (*T_sec air_*)	330 °C
Secondary air temperature at the inlet of air preheater (*T_sec air,in_*)	23 °C
Secondary air flow rate ratio (*α_sec_*)	81%
Excess air coefficient (*α_air_*)	1.2
Exit flue gas temperature (*T_fg, ex_*)	123 °C
Environment temperature	20 °C
Pinch temperature between *T_fg_*_,4_ and *T*_4_ (*∆T_p,_*_4_)	40 °C
Pipeline efficiency (*η_p_*)	99%
Power generator efficiency (*η_g_*)	98.5%

**Table 2:** Reference bottom cycle parameters.

Variable/Parameter	Values
Turbine inlet temperature (*T*_5*b*_)	400–600 °C
Turbine inlet pressure (*P*_5*b*_)	15–45 MPa
Turbine isentropic efficiency (*η_t,s_*)	93%
Compressor inlet temperature (*T*_1*b*_)	32 °C
LP compressor inlet pressure (*P*_1*b*_)	7.6 MPa
Compressors isentropic efficiency (*η_c,s_*)	89%
Pressure drop of each component except the boiler (*ΔP*)	0.1 MPa
Pressure drop of the boiler (*ΔP_b_*)	0.2 MPa
LTR2 and HTR2 pinch temperature difference (*ΔT_LTR_*_2_ or *ΔT_HTR_*_2_)	10 °C

**Table 3:** Properties of the designed coal.

C*_ar_*	H*_ar_*	O*_ar_*	N*_ar_*	S*_ar_*	A*_ar_*	M*_ar_*	V*_daf_*	Q*_f_*
61.70	3.67	8.56	1.12	0.60	8.80	15.55	34.73	23442

C (carbon), H (hydrogen), O (oxygen), N (nitrogen), S (sulfur), A (ash), M (moisture), V (volatile). Subscripts *ar*, *daf* means as received, dry and ash free, *C_ar_* + *H_ar_* + *O_ar_* + *N_ar_* + *S_ar_* + *A_ar_* + *M_ar_* = 100.
